# Structure of executive functions in young and in older persons

**DOI:** 10.1371/journal.pone.0216149

**Published:** 2019-05-09

**Authors:** Otmar Bock, Mathias Haeger, Claudia Voelcker-Rehage

**Affiliations:** 1 Institute of Physiology and Anatomy, German Sport University, Köln, Germany; 2 Institute of Human Movement Science and Health, Technical University Chemnitz, Chemnitz, Germany; Monash University, AUSTRALIA

## Abstract

**Introduction:**

Using factor analysis, several studies reported that higher-order cognitive control involves separable executive functions. However, the number and definition of the purported functions differed between studies. One possible explanation for this discrepancy is that executive functions don’t exhibit a clear factorial structure, i.e., there is no clear dichotomy between executive function tests which are well-correlated (representing a common factor) and those which are poorly correlated (representing distinct factors). We scrutinize this explanation separately in data from young and from older persons.

**Methods & results:**

Young and older volunteers completed cognitive tests of the purported executive functions shifting, updating, inhibition and dual-tasking (two tests per function). Confirmatory and exploratory factor analyses yielded, for either age group, factorial structures that were within the range reported in literature. More importantly, when correlations between tests were sorted in ascending order, and were then fitted them by piecewise linear regression with a breakpoint, there was no evidence for a distinct breakpoint between low and high correlations in either age group. Correlations between tests were significantly higher in older compared to young participants, and the pattern of test pairs with high and with low correlations differed between age groups.

**Discussion:**

The absence of a breakpoint indicates that executive function tests don’t segregate into well-correlated and poorly correlated pairs, and therefore are not well suited for factor analyses. We suggest that executive functions are better described as a partly overlapping rather than a factorial structure. The increase of correlations in older participants supports the existence of age-related dedifferentiation, and the dissimilarity of correlations in the two age groups supports the existence of age-related reorganization.

## Introduction

It has been proposed decades ago that human cognition is coordinated and supervised by a higher-order mechanism, probably residing in the frontal cortex [1,2]. This mechanism has later been formalized as “supervisory attention system” [3] or “central executive” [4]. Later authors argued that a monolithic supervisory mechanism is nothing more than a “homunculus” with little explanatory value [5,6] and instead proposed the existence of multiple supervisory processes, often under the umbrella term ‘executive functions’. As an example, one of the most influential studies in this field stipulated three executive functions, ‘updating of working memory contents’, ‘shifting between tasks or mental sets’ and ‘inhibition of prepotent responses’ [7].

To establish their concept, Miyake et al. [7] asked participants to complete three cognitive tests which quantified their ability for updating, three which assessed shifting and three which registered their ability for inhibition. The resultant test scores were submitted to confirmatory factor analyses (CFAs), a technique which determines the goodness-of-fit between experimental data and a pre-established factorial model. The authors compared several alternative models and found the best fit with a model that consisted of three mutually correlated factors, one associated with tests on updating, one with those on shifting and one with those on inhibition.

It is important to note that CFA-based approaches have a propensity for self-fulfilling prophecy. They can determine which among several pre-established factorial models fits the available data best, but they are unable to derive a new, even-better-fitting model. Miyake et al. [7] overcame this problem by calculating not only CFAs, but also an exploratory factor analysis (EFA). This approach is less susceptible to self-fulfilling prophecy since it calculates the best-fitting factorial model without recourse to any preconceived model. EFA yielded three mutually correlated factors as well, one closely associated with tests on updating, one with those on shifting and one with those on inhibition. This outcome therefore seems to support the existence of the three executive functions stipulated by Miyake et al. [7]. However, the same author group later presented an alternative factorial model, which replaced ‘inhibition’ by ‘common executive function’, i.e., by a factor that was associated with all nine cognitive tests [8].

Participants of Miyake et al. [7] also completed a test of dual-tasking. The scores on that test were not associated significantly with any of the three CFA factors; it therefore has been suggested that dual-tasking may represent a distinct executive function [9]. Accordingly, some recent studies included dual-tasking as a fourth executive function (cf. [10,11]). Yet other studies stipulated alternative executive functions such as ‘rule detection’, ‘concept formation’, ‘strategic planning’, ‘estimation’ and ‘emotional control’ (cf. [12]).

Summing up, earlier research presented conflicting views about the factorial structure of executive functions. One possible explanation for this disagreement is that factor analyses, in general, are not well suited for the data at hand. Simply speaking, factor analyses aggregate well-correlated variables to a common factor and segregate poorly correlated variables to distinct factors; this works well if some of the correlations are markedly higher than others, but it becomes problematic if there is no clear dichotomy between high and low correlations. Without such a dichotomy, even small fluctuations of the correlation pattern could substantially modify the best-fitting factorial model, which could explain the discrepancies in literature. A glance at published correlations between executive-functions [13–15,7] reveals no easily discernible dichotomy. One purpose of the present study was, therefore, to scrutinize this observation in a quantitative fashion.

Above research on executive functions dealt with young participants, leaving open how the structure of those functions changes with advancing age. It is well established that older persons perform less well than young ones on a range of executive function tests (cf. meta-analyses by [16–18]([19,20], but poorer performance as such does not necessarily imply a changed factorial and/or correlational structure. However, that structure could be affected by two other age-related phenomena. One of them is cognitive dedifferentiation, which manifests as increased correlation between the performance scores on different cognitive tasks [21,22]. Age-related dedifferentiation probably results from biological decay, cultural influences and lifelong experience [23–25], and corresponds at the neuronal level to the recruitment of larger and more overlapping brain areas for a given task [26]. The other phenomenon is cognitive reorganization, i.e. the engagement of different cognitive processes [27] and brain areas [28] for the same cognitive task. Both phenomena are likely to impact the structure of executive functions: age-related dedifferentiation should reduce the number of factors and/or increase the correlations between test pairs, while age-related reorganization should decrease the correlations between some test pairs and might increase those between some other test pairs.

Several studies used CFA-based approaches similar to Miyake et al. [7] to evaluate the structure of executive functions in healthy older adults. In one study [29], the best fitting model had the same tri-factorial structure as that of Miyake et al. [7]. Four other studies yielded best fits with bi-factorial models which combined updating and shifting [30,15] or updating and inhibition [31] to a single factor, or stipulated the executive functions ‘working memory’ and ‘access to long-term memory’ [13]. Finally, one study reported the best fit with a single-factor model [14]. Taken together, these studies tell us little about the structure of executive functions in older compared to young adults. The number and meaning of factors varied between studies, as they did in research with young participants, and consistent differences between age groups are therefore difficult to identify. To get a clearer picture about age-related differences, it might be helpful to administer executive function tests to young and older persons in one and the same study, thus eliminating differences between the selected tests, instructions, settings and experimenters’ personalities.

We are aware of only two factor analytical studies which took such an approach. One of them used CFA and yielded a model with two factors [13], while the other calculated EFA and yielded a model with four factors [32]. Importantly, both studies calculated common models for participants of all ages and treated ‘age’ as a mediating variable. As a consequence, both studies accounted for the effects of age on factor loadings, but neglected the effects of age on the factorial and/or correlational structure of executive functions. For example, if young persons were best characterized as having four but older ones as having two executive functions, the common models calculated by both studies would be oblivious of this fact. The second purpose of the present study was, therefore, to find out whether the structure of executive functions is the same for young and for older persons. To our knowledge, no study has done this before. We expected that in older age, correlations between test pairs will be generally higher because of age-related dedifferentiation, and the pattern of relatively high and relatively low correlations will be different because of age-related reorganization.

Participants’ performance on executive function tests is often quantified in terms of reaction time. However, this metric may be biased since it doesn’t account for the existence of a speed-accuracy tradeoff: when responding in a cognitive test, we can be quick at the expense of accuracy or accurate at the expense of speed [33,34]. This is particularly relevant for studies which compare young and older individuals, since older persons place a stronger emphasis on accuracy [35,36]. An age-related increase of reaction time may therefore reflect not only a deficit of the targeted cognitive function, but also a shift of emphasis from fast to accurate responses.

A second bias may arise because reaction time is sensitive not only to the speed of the targeted cognitive function, but also to the speed of upstream sensory and low-level cognitive processes [37]. Since processing speed generally decreases in older age [38], an age-related increase of reaction time may reflect such generalized slowing rather than a specific deficit of the targeted cognitive function. It has indeed been shown that the decay of executive functions in older age is partly attributable to decreasing psychomotor speed [39–41].

It is unknown to what extend the two phenomena, shifts of the speed-accuracy tradeoff and generalized slowing, are mutually independent. We therefore decided to control for both in our study.

## Methods

### Participants

Sixty-three young (age 20–30 years; M = 23.17, SD = 2.83, females = 40) and 61 older adults (age 65–75 years; M = 69.97, SD = 2.96, females = 22) volunteered to participate in a larger research project on various aspects of cognitive aging, of which the present study was a smaller part. They were recruited by paper and electronic postings, and by contacts with local senior networks in Köln and in Chemnitz. 39 young and 29 older participants were tested in Köln and the remaining ones in Chemnitz, using the same hard- and software and the same formalized instructions. All participants were in good physical and mental health by self-report, had no history of stroke or brain surgery, and their visual acuity—as assessed by the Freiburg Vision Test (FrACT version 3.9.0)—was better than 20/60, which is sufficient for safe car driving [42]. Screening tests ensured that participants didn’t suffer from cognitive impairment (Mini Mental State Examination > 26 points), from language comprehension deficits (Freiburger Sprachverständlichkeitstest > 50% word recognition at best hearing level) or obesity (cutoff: BMI ≥ 30). The Edinburgh Handedness Inventory [43] revealed that five participants were left-handed, one was ambidextrous but used the right hand for typing, and all others were right-handed. Persons who wore contact lenses, prescription glasses or hearing aids in everyday life did so as well during the tests. This study was carried out in accordance with the Declaration of Helsinki. All participants signed a written informed-consent statement. The research protocol was pre-approved by the Ethics Commission of the German Sport University (approval # 27/2015). Participants received a compensation of 60€ in total.

### Tests

We programmed a battery of executive function tests using E-Prime 2.0. Stimuli were presented on a 24” computer screen and through loudspeakers. Each test was preceded by a standardized instruction display, and by up to three practice trials. The rationale for selecting those particular tests was to replicate the three-factor-plus-dual-tasking model of Miyake et al. [7], since this is one of the most influential models in literature (cf. Introduction).

*Updating* was assessed by a keep-track test and an n-back test, both adapted from literature [7,44,45]. In the former test, 15 words from six different categories (animals, colors, relatives, metals, countries, distances) were displayed on the screen in a randomized sequence for 2000 ms each, with an inter-stimulus interval (ISI) of 800 to 1200 ms. Participants were instructed to attend to three of those categories and, after presentation, to write down the last word from each of those three categories. Then they pressed the “M” key of a keyboard with their right index finger to start the next trial, which presented 15 new words from the same six categories. There was a total of six trials, and the number of categories to report changed from trial to trial in the order 3,3,4,4,5,5. Outcome measure was the percentage of correct responses across all trials.

In the n-back test, a 4x4 grid was displayed continuously on the screen. Black dots were sequentially presented in the center of different grid cells for 500 ms each, for a total of 19 dot presentations. ISI was again 800 to 1200 ms. Participants had to press the “M” key within their right index finger if the currently displayed dot was in the same position as the second-to-last dot, and otherwise to press the “X” key with their left index finger. The test consisted of six 19-dot trials, with a pause of five seconds between trials (except after trial 3, where the pause was 20 s). Outcome measures were the percentage and mean latency of correct responses across all trials, omitting the first two dots of each trial where no n-back response could be given. Responses outside a time window of 2000 ms after stimulus onset were considered as ‘incorrect’.

*Shifting* was assessed by two task switching paradigms, again modified from previous work [46,47]. In test switch-semantic, 17 words were sequentially displayed in the center of the screen for 1500 ms each, with an ISI of 800 to 1200 ms. A central fixation cross was displayed during each ISI. Participants had to press either the “M” key with their right or the “X” key with their left index finger, to indicate whether the current word was mono- or bi-syllabic (subtask A), or whether it denoted an inanimate or a living object (subtask B). The order of subtasks on each 17-word trial was AABBAABBAABBAABBA, without external cues about subtask order [20]. The test consisted of six trials, with pauses as in the n-back test. Outcome measures were the percentage of correct responses and the switching costs across all trials. Switching costs were calculated as the difference between the mean latency of correct responses after a subtask switch minus that after a subtask repetition, discarding the first stimulus on each trial. Responses outside a time window of 2000 ms after stimulus onset were considered as ‘incorrect’.

Test switch-spatial was similar, except that words were replaced by geometrical shapes and participants had to discriminate between circular and quadratic shapes (subtask A) or between big and small shapes (subtask B).

*Inhibition* was registered by a Simon and a Stroop test, both adapted from literature [48–50]. In the Simon test, 32 left- or rightward pointing arrows were sequentially presented to the left or right of the screen center for 500 ms each, with an ISI of 800 to 1200 ms. Arrow presentation again alternated with a central fixation cross. On one-half of the trials, position and direction of the arrow were compatible; on the other half, they were incompatible. Participants had to depress the “M” key with their right index finger if the arrow pointed to the right, and the “X” key with their left index finger if the arrow pointed to the left, irrespective of the arrow’s position. Outcome measures were the percentage of correct responses and the inhibition costs across all trials. Inhibition costs were calculated as the difference between the mean latency of correct responses to incompatible stimuli minus that to compatible stimuli. Responses outside a time window of 2000 ms after stimulus onset were considered as ‘incorrect’.

In the Stroop test, 32 color-denoting words were sequentially presented in the center of the screen for 500 ms each, with an ISI of 2300 to 2700 ms. Again, stimuli alternated with a central fixation cross. One-half of the words were displayed in a color that was compatible with the word’s meaning, red, green, yellow or blue. The other half was displayed in an incompatible color. Two response words were displayed below each target word for 2000 ms, one somewhat to the right and the other somewhat to the left. Both response words were white; one named the color of the target word (i.e. correct response) and the other named one of the other colors. The position (left/right) of the correct response word was randomized across trials. Participants had to depress the key that was closest to the correct response: either the “M” key with their right or the “X” key with their left index finger. Outcome measures were as in the Simon test.

*Dual-tasking* was quantified by a combination of manual tracking and tone monitoring, again adapted from literature [51]. In the tracking test, a red target square moved across the screen from left to right along an unpredictable path; vertical position represented the sum of six sinewaves, and sweep time was 45 s. Participants tracked the target with a joystick-driven cross, and their performance was quantified as root mean square error (RMSE). In the tone monitoring test, ten high-pitched (1086 Hz), ten middle-pitched (652 Hz) and ten low-pitched (217 Hz) tones were presented in a random sequence from the beginning until the end of each trial. Participants had to respond to the high-pitched tone only, either by depressing the “M” key with their right index finger (manual monitoring) or by uttering “yes” (verbal monitoring). Performance was quantified as accuracy and latency of correct responses. Only responses given before the onset of the subsequent tone were considered for this analysis.

Each participant performed the tests one day with manual monitoring, and another day with verbal monitoring (mixed order). Each day began with a practice trial of tracking with concurrent monitoring. Then came nine experimental trials: a block of three tracking-only trials, a block of three monitoring-only trials, and a block of thee tracking trials with concurrent monitoring. The order of blocks was balanced across participants. Outcome measures were the dual-task costs of tracking ([RMSE(dual)–RMSE(single)] / RMSE(single)), the percentage of correct monitoring responses, and the dual-task costs of monitoring ([latency(dual)–latency(single)] / latency (single)). These measures were calculated once for the combination of tracking with verbal monitoring (‘tracking-verbal’ for short) and once for the combination of tracking with manual monitoring (‘tracking-manual’ for short).

*Psychomotor speed* was assessed by a manual tapping test. Following established procedures [52], participants placed their non-dominant hand on a table and tapped with their dominant hand back and forth across the non-dominant hand. The time to complete 25 full tapping cycles was registered by a stopwatch. The test consisted of three trials, and outcome measure was the shortest registered time.

As already mentioned, this study was part of a larger research project on various aspects of cognitive aging. Each participant completed four experimental sessions on separate days, with at least one day off in-between. This took between 8 and 28 days, depending on the participants’ availability. Before the first session, participants completed at home a set of questionnaires regarding their sex and age, education (highest school degree, years of formal education), health (overall quality, days sick per year, accidental falls per year, falls efficacy as per FES), physical activity (hours of moderate and of strenuous physical activity per week), car use (km driven per year) and social activity (type and frequency). The first session included screening tests and tapping; the remaining three sessions included the above executive function tests, in an order that was balanced across participants. In each given session, executive function tests were administered prior to any other tests.

### Data analysis

Outliers were eliminated from latency data using the ±3.29 SD criterion [53], separately for each participant and variable. Data were then averaged across repetitions, again separately for each participant and variable. Participants with accuracy <0.60 on any test except *keep track* were classified as random performers and were excluded from further analyses. (We did so because all tests except *keep track* had two response alternatives, i.e. random responses would have an accuracy of 0.5; to be on the safe side, we selected 0.6 as rejection criterion. In case of *keep-track*, we reasoned that random performers who remember all presented words will be correct on 1/455 trials, 1/368 trials and 1/286 trials when picking three, four and five words, respectively, which corresponds to an accuracy of 0.0028. Obviously, participants are not likely to remember all 15 presented words due to WM capacity limitations, and the words they remember may include all, some or none of the target words; however, accuracy of random performance will still be in the same order of magnitude as calculated above. Since the lowest *keep-track* accuracy in our study was 0.25, we decided not to exclude participants because of their *keep-track* accuracy.) Data from the other participants were converted into q-scores to control for speed-accuracy tradeoffs, using [54]

$$q=\frac{z(LAT)*15+100}{z(ACC)*15+100},$$ (1)

where z(LAT) is the mean latency standardized across participants irrespective of their age, and z(ACC) is the corresponding standardized mean accuracy. Note that by calculating q-scores across both age groups, any differences between young and older persons were preserved. Note further that higher q-scores indicate poorer performance. Three special cases emerged when applying Eq (1). First, data from the *keep-track* test and from the tracking part of both dual-tasks included no latencies; we therefore decided to calculate the pertinent q-scores with the numerator of Eq (1) set to 100, i.e., to the mean numerator value of the other tests. Second, tracking performance on both dual tasks was quantified as error rather than accuracy; to account for this fact, we entered the error rather than accuracy into Eq (1) and inverted the sign of the resultant q-scores. Third, each dual-task yielded two q-scores, one for tracking and one for task monitoring; in accordance with earlier dual-task research [55,56], we used the mean of both scores for further analyses.

Most of our analyses build upon the bivariate correlations between q-scores from different tests. Since correlations are sensitive to outlying residuals, we identified such outliers by a procedure adopted from Miyake et al. [7]. We calculated Cook’s D scores for each test pair, and excluded all individuals with D>1 on any test pair. D>1 is an established criterion for the identification of outlying residuals [57]. Data from the remaining participants were submitted to the following analyses.

A first set of analyses addressed the predictive value of our home-based tests for participants’ scores on the executive function tests. To this end, we used multiple stepwise linear regression analysis with the following regressors: calendric age, sex, years of formal education, days sick per year, accidental falls per year, falls efficacy, hours of moderate physical activity per week and hours of strenuous physical activity per week. This analysis was run separately with the q-scores from each executive function test as dependent variable. The following variables were not included as regressors since too many scores were zero: number of days sick and number of falls. The following variables were not included as regressors since they were ordinally scaled: overall health quality, car use and social activity. Instead, the latter three variables served as factors for an analysis of co-variance (ANCoVA), co-variates were the significant regressors from above stepwise regression analyses. ANCoVA was run separately with the q-scores from each executive function test as dependent variable.

Subsequent analyses dressed the factorial structure of q-scores. We calculated CFA with four correlated factors; factor ‘updating’ was linked to *n-back* and *keep-track*, factor ‘shifting’ to *switching-spatial* and *switching-semantic*, factor ‘inhibition’ to *Simon* and *Stroop*, and factor ‘dual-tasking’ to *tracking-verbal* and *tracking-manual*. This CFA model is analogous to the best fitting model of Miyake et al. [7], except that dual-tasking is treated as a factor rather than as an external variable. Goodness-of-fit was assessed by a χ^2^-test. CFA was calculated once with the data from young, and once with those from older participants.

We also calculated EFA, using principal component extraction with a minimum eigenvalue of 1 and with standardized varimax rotation. Again, this was done separately for the data of young and those of older participants. In accordance with literature [58], factor loadings >0.7 were deemed to be “satisfactory”.

The focus of our analyses was not on CFA and EFA, but rather on the underlying bivariate correlations between executive function tests. Our eight tests could be combined to 28 different test pairs, and we therefore yielded 28 test pair correlations for young and 28 for older participants. We sorted the correlations from each age group in ascending order, fitted them by piecewise linear regression with a single breakpoint [59], and used the resultant regression parameters to calculate the metric

$${(c}_{post}-c_{pre})-{(c}_{pre}-c_{1}).$$ (2)

In Eq (2), c_1_ is the predicted lowest correlation of the ascending sequence, c_pre_ is the predicted last correlation before the breakpoint and c_post_ is the first one after the breakpoint. We decided to accept the existence of a distinct breakpoint if the above metric returns a positive value, i.e., if correlations change across the breakpoint more than they do to the left of it.

In further analyses, test pair correlations of either age group were tested against zero and against each other using t-tests. Since correlations are not normally distributed in smaller samples [60], we first transformed them to Fisher’s Z (not to be confused with a z-transformation, as used for standardizing), performed the t-tests, and then back-transformed the calculated means and standard deviations for reporting.

In a final analysis, we calculated the linear regression of older persons’ correlations on young persons’ correlations. If the pattern of test pairs with high and with low correlations is similar in both age groups, the regression slope should be significant.

## Results

Table 1 lists the number of participants excluded from analysis. By far the most frequent reason for exclusion was random test performance by older persons. Fifty-nine young persons (23.15 ±2.91 years old, 20 males) and forty-two older persons (69.95 ±2.94 years old, 26 males) remained for further analyses. Table 2 summarizes the data collected from home-based questionnaires for those remaining participants: older persons were somewhat less physically active, less likely to suffer an accidental fall, less often sick, and more likely to drive car over longer distances.

**Table 1:** Exclusion of participants.

exclusion based on:	random performance	outliers	total
young adults	3	1	4
older adults	15	4	19

**Table 2:** Demographics and outcome of home-based questionnaires in young and in older participants.

	young persons	older persons
number of females / number of males	39 / 20	16 / 26
age (mean ±SD)	23.15 ±2.91	69.95 ±2.94
school education level*	high school diploma	high school diploma
years of formal education (mean ±SD)	15.4 ±2.4	15.6 ±2.6
health quality*, **	good	good
days sick per year (mean ±SD)	10.9 ±8.4	3.4 ±6.0
accidental falls per year (mean ±SD)	0.102 ±0.443	0.048 ±0.216
falls efficacy (mean ±SD)***	16.7 ±1.0	16.7 ±2.4
hrs/week moderate physical activity^§^	14.3 ±14.9	12.6 ±13.7
hrs/week strenuous physical activity^§^	6.1 ±6.3	4.5 ±4.2
km/year car use*	<6000	9000–12000
number of social activities per month*,^§§^	18	18

* Responses were on an ordinal scale; we report the most frequently checked response bin.

** Five response alternatives, ranging from “poor” to “very good”.

*** Scores range from 16 = no concerns on any item to 64 = strong concerns on all items.

^$^ Includes sports, leisure and work

^§§^ Indicated on a list of 18 common activities such as concerts, parties, care of dependent persons

**Fig 1:**
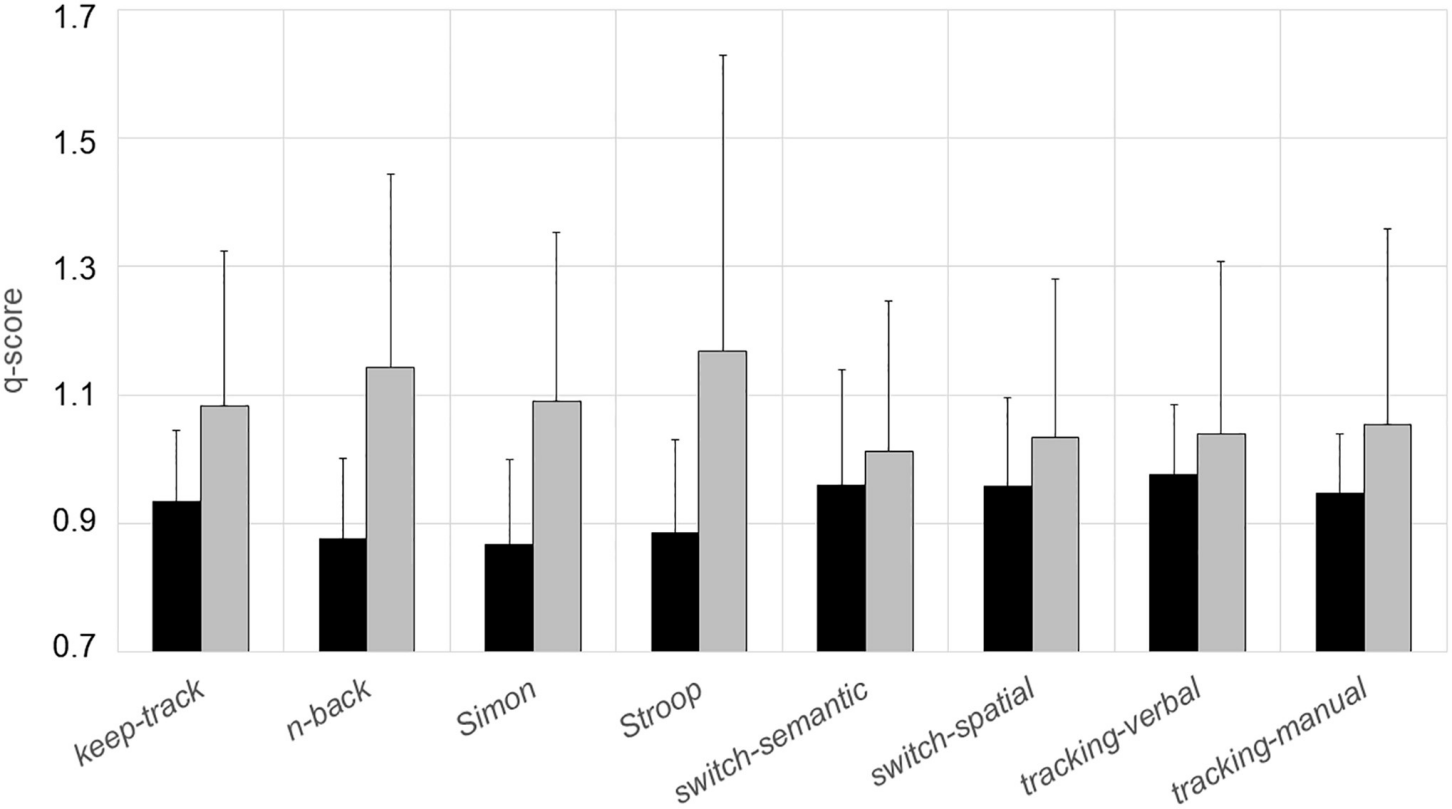
Mean value of q-scores in each executive function test. Data from young participants are shown in black, and those from older participants in gray. Error bars are standard deviations.

Fig 1 plots the mean q-scores of individuals that remained for further analyses. Older persons had higher q-scores (i.e., poorer performance) on all tests: the age difference ranged from 8.5% in *switch-semantic* to 36.1% in *Stroop*.

Table 3 summarizes the outcome of stepwise multiple linear regressions. q-scores from *all* executive function tests were significantly related to calendric age (higher age ~ poorer performance). q-scores from *some* tests were significantly related to years of education (more years ~ better performance), falls efficacy (more concerns ~ better(!) performance) and hours of moderate physical activity (more activity ~ poorer(!) performance). Notably, q-scores from *no* test were significantly related to sex or psychomotor speed. We decided to use only calendric age as co-variate for the subsequent ANCoVA, since only calendric age had strong and meaningful associations with all executive function tests. We further decided to proceed with data analyses without correcting for sex and psychomotor speed.

**Table 3:** Outcome of stepwise multiple linear regression analyses*.

	regressor			
test	calendric age	education years	falls efficacy	moder. phys. act.
*keep-track*	**0.494**	n.s.	**-0.434**	n.s.
*n-back*	**0.622**	-0.204	n.s.	n.s.
*Simon*	**0.628**	-0.214	-0.225	n.s.
*Stroop*	**0.387**	n.s.	-0.215	0.224
*switch-semantic*	0.207	n.s.	-0.233	n.s.
*switch-spatial*	**0.342**	n.s.	n.s.	n.s.
*tracking-verbal*	0.246	n.s.	n.s.	n.s.
*tracking-manual*	**0.350**	n.s.	n.s.	n.s.

*Note: “moder. phys. act” refers to the time spent on moderate physical activity. Cell entries are partial correlations between regressors and q-scores. Bold font indicates p<0.001, regular font indicates p<0.05 and n.s. indicates p>0.05. Not shown are regressors which yielded no significant partial correlations with any executive function test.

ANCoVA yielded no significant effects on any executive function test for the factors health status (all F < 1.31; all p>0.05) and social activity (all F<2.46; all p>0.05). Car use had a significant effect on the q-scores from *n-back* (F(4,64) = 2.96; p <0.05) and from *tracking-manual* (F(4,64) = 3.05; p <0.05).

The outcome of CFA with the pre-established four-factor model (see Methods) is presented in Table 4. Most but not all factor loadings reached statistical significance, and the model as a whole fitted reasonably well the q-scores of young (χ^2^(14) = 22.45; p>0.05) as well as to those of older adults (χ^2^(14) = 14.59; p>0.05). Correlations between factors ranged from -0.11 to 0.32 in young and from -0.12 to 0.64 in older persons.

**Table 4:** Outcome of confirmatory factor analyses for young and for older persons. Note: Cell entries are the loadings of a given test (2^nd^ column) on a given factor (1^st^ column), as well as the pertinent standard errors, values of the asymptotic normal statistic and p-values. Bold p-values indicate statistical significance. The asymptotic normal statistic is the probability distribution for loading = 0.000.

CFA factor	test	loading	S.E.	ANS	p	loading	S.E.	ANS	p
updating	*keep-track* *n-back*	0.111	0.010	10.77	**<0.001**	0.185	0.020	9.055	**<0.001**
		0.010	0.017	0.60	>0.05	0.013	0.038	0.338	>0.05
inhibition	*Simon* *Stroop*	0.045	0.017	2.67	**<0.01**	0.076	0.036	2.126	**<0.05**
		0.145	0.013	10.77	**<0.001**	0.330	0.095	3.474	**<0.01**
switching	*switch-semantic* *switch-spatial*	0.181	0.017	10.77	**<0.001**	0.181	0.020	9.055	**<0.001**
		0.039	0.018	2.16	**<0.05**	0.047	0.029	1.601	>0.05
dual-tasking	*tracking-verbal* *tracking-manual*	0.110	0.010	10.77	**<0.001**	0.074	0.033	2.245	**<0.05**
		0.027	0.012	2.30	**<0.05**	0.261	0.029	9.055	**<0.001**

The outcome of EFA is listed in Table 5. Four factors emerged from the data of young persons; they largely correspond to the four purported executive functions, inhibition, shifting, dual-tasking and updating, although two of the eight tests had no satisfactory (> 0.7) loadings. Only three factors emerged from the data of older persons, none of them clearly associated with a presumed executive function. In this age group, four of the eight tests had no satisfactory loadings. Taken together, factors explained 68.4% of total variance in young and 61.2% of total variance in older persons.

**Table 5:** Outcome of exploratory factor analyses for young and for older persons.

	young persons				older persons		
test	F1	F2	F3	F4	F1	F2	F3
*keep-track*	-0.053	-0.042	0.039	**0.925**	**0.815**	0.031	0.010
*n-back*	-0.550	0.194	0.031	0.198	0.155	0.172	**-0.815**
*Simon*	**0.760**	0.141	0.036	-0.013	0.588	0.240	-0.183
*Stroop*	**0.720**	-0.036	0.018	0.477	0.424	0.645	0.151
*switching-semantic*	0.169	**0.873**	0.218	-0.115	**0.815**	-0.234	0.227
*switching-spatial*	-0.323	0.644	-0.274	0.073	0.214	0.249	0.619
*tracking-verbal*	0.085	0.277	**0.769**	0.245	-0.127	0.623	0.072
*tracking-manual*	-0.061	-0.174	**0.801**	-0.119	0.045	**0.868**	-0.151
variance expl’d.	0.194	0.168	0.170	0.152	0.243	0.220	0.148

Note: Cell entries are the loadings of a given test (1st column) on a given factor (2^nd^ row). Loadings >0.7 are highlighted in bold.

**Fig 2:**
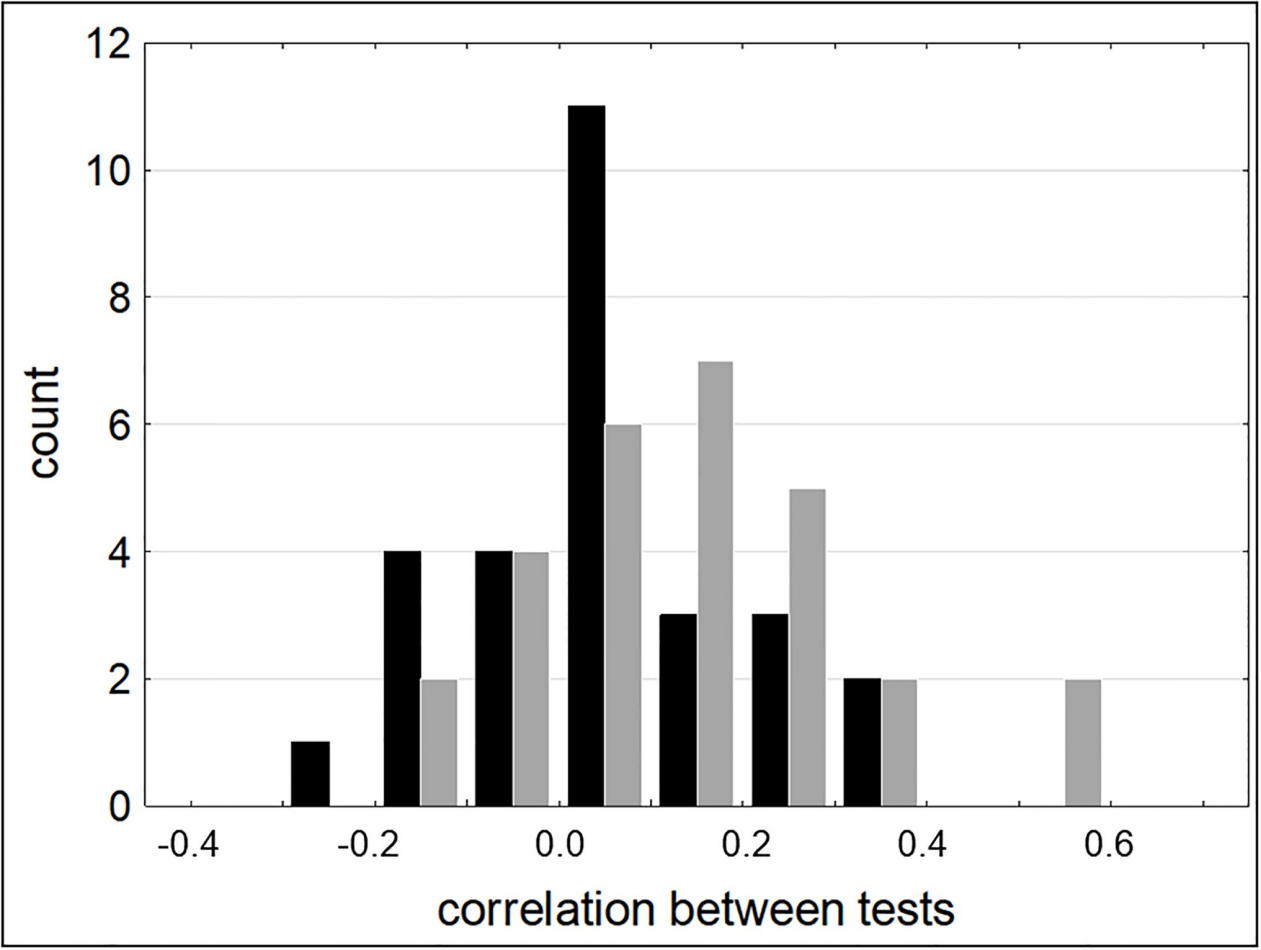
Frequency distribution of bivariate correlations between test pairs. Data are plotted separately for young (black) and for older participants (gray). For example, two correlations in older participants were in the range 0.5 to 0.6.

**Fig 3:**
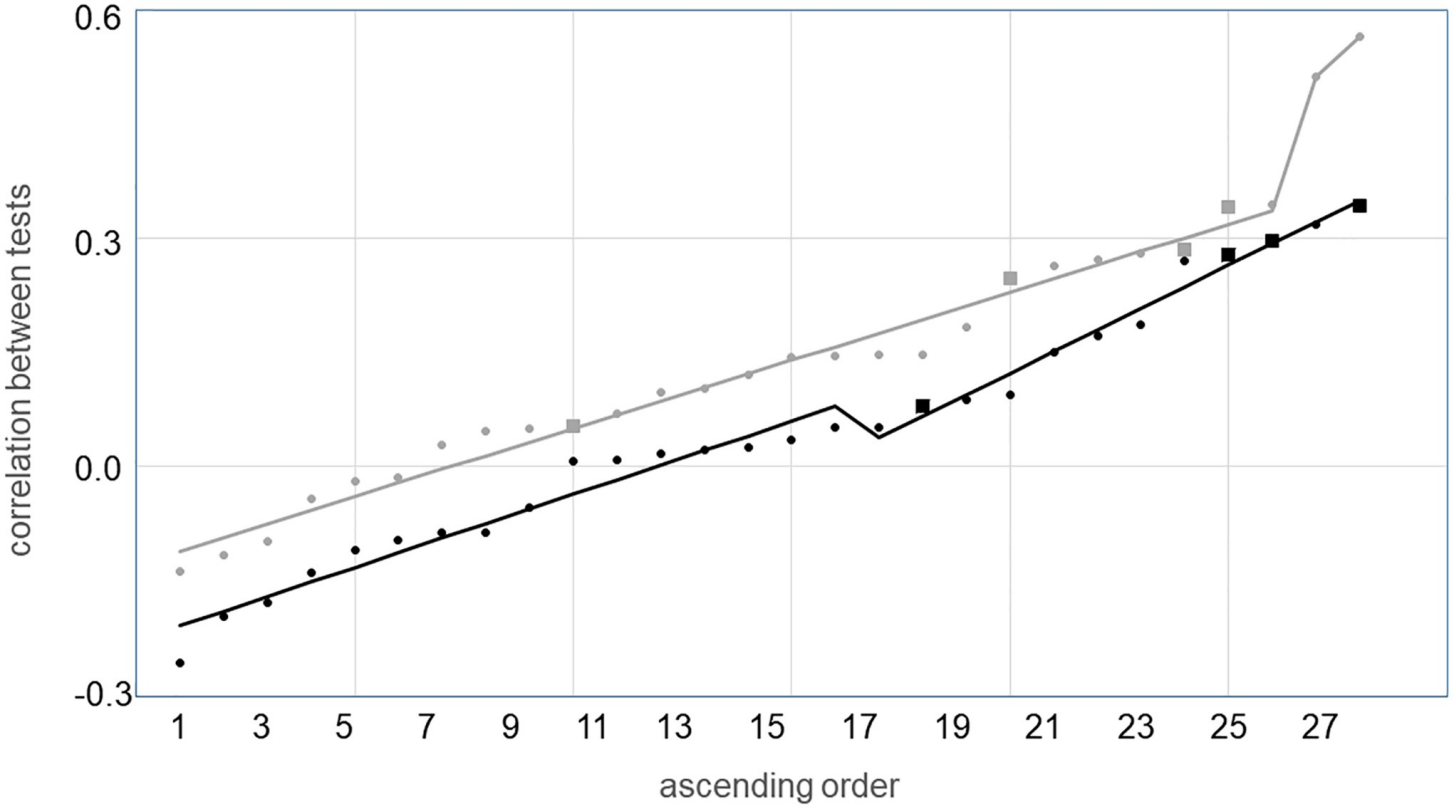
Bivariate correlations between test pairs, plotted in ascending order. Data from young participants are shown in black, and those from older participants in gray. Negative correlations imply that good performance on one test was associated with poor performance on the other test. Large squares denote test pairs which purportedly represent a common executive function. From bottom left to top right, the squares stand for updating, shifting, dual-tasking and inhibition in young persons, and for updating, shifting, inhibition and dual-tasking in older ones. Solid lines are predictions of piecewise linear regression with one breakpoint.

Fig 2 shows the frequency distribution of test pair correlations, separately for young and for older persons. No conspicuous dichotomy between a cluster of lower and a cluster of higher correlations can be discerned in the distribution for either age group. Fig 3 plots the same correlations individually, sorted in ascending order. It is interesting to note that correlations between test pairs which purportedly represent the same executive function (lager symbols in Fig 3) are not necessarily high. Lines in Fig 3 depict the outcome of piecewise linear regressions, and the pertinent regression parameters are listed in Table 6. According to that table, our criterion for the existence of a distinct breakpoint (see above), was not met by the data from either age group.

**Table 6:** Outcome of piecewise linear regression of test pair correlations in young and older persons.

	IC1	SL1	IC2	SL2	order	R	c_post_ − c_pre_	c_pre_ − c_1_	DB
young	-0.129	0.018	-0.905	0.052	26	0.994	-0.041	0.228	no
older	-0.229	0.019	-0.444	0.028	16	0.992	0.176	0.447	no

Note: IC1, IC2, SL1 and SL2 are the y-intercepts and slopes of the 1^st^ and 2^nd^ linear segment, respectively. Order is the serial order of the last test pair correlation before the breakpoint, and R quantifies the goodness-of-fit of piecewise linear regression. The two subsequent columns list quantities defined in Eq (2) and the rightmost column indicates whether our criterion for the existence of a distinct breakpoint is met by the data.

**Fig 4:**
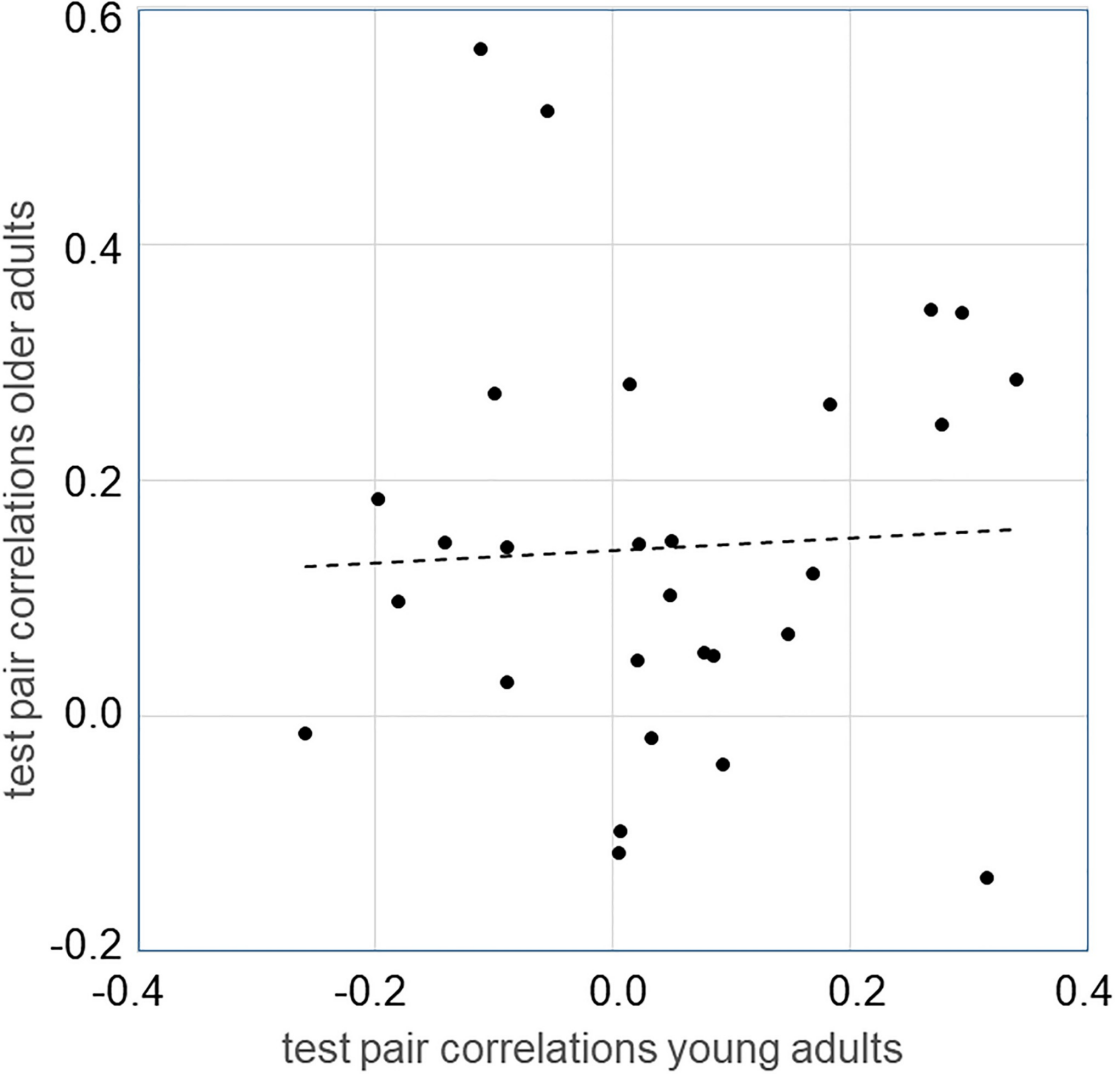
Pattern of test pair correlations in the two age groups. Each symbol represents the correlation for one test pair in older persons, plotted against the correlation for the same test pair in young persons. Dashed line is the regression line.

The mean and standard deviations of test pair correlations were 0.047 ±0.164 in young, and 0.149 ±0.185 in older persons. Young participants’ scores were not significantly different from zero (t(27) = 1.47; p>0.05), but they were significantly different from older participants’ scores (t(54) = 2.175; p<0.05). Fig 4 illustrates once more the test pair correlations already shown in Figs 2 and 3, this time comparing the data of young and older participants for a given test. Linear regression of the data in Fig 4 yielded a slope of 0.045, which is not statistically significant (t(26) = 0.244, p>0.05).

## Discussion

The present study examined the structure of executive functions in young and older persons. We found that older participants performed less well than young ones on a range of executive function tests, that bivariate correlations between test pairs failed to segregate into relatively high and relatively low correlations in either age group, and that correlations were significantly higher in older persons than in young ones.

We found no strong and consistent effects of sex, education, physical activity and health on our participants’ executive functions, possibly because our sample was generally healthy and fit (cf. Table 2). The observed effect of age is in agreement with numerous earlier studies (cf. meta-analyses [16,19,17,18,20]. However, earlier work found that this decay is partly attributable to decreases of psychomotor speed [39–41], which was not the case in our data. This apparent discrepancy is probably due to methodological issues: earlier work quantified executive performance mainly in terms of response latency, thus disregarding the age-related shift of priority from speed to accuracy [35,36], while the present study used q-scores, thus taking speed-accuracy tradeoffs into account. It therefore is conceivable that shifts of priority and decreases of psychomotor speed in older age are related phenomena. Since we found no effects of psychomotor speed on q-scores, we disregarded psychomotor speed in subsequent analyses.

Our participants’ q-scores were only in part compatible with a four-factor model, consisting of the three factors postulated by Miyake et al. [7] plus the factor ‘dual-tasking’. In a confirmatory factor analysis, data from either age group were not significantly different from that model’s predictions. However, one test in young and two in older persons achieved only negligible factor loadings. In an exploratory factor analysis, data from young persons yielded a four-factor solution reminiscent of the above model while data from older persons yielded a three-factor solution less clearly related to that model; two tests in young and four in older persons achieved only unsatisfactory (<0.7) factor loadings. This outcome complements the discrepant executive-function models available in literature, where the number and definition of factors differ from study to study, both for young and for older persons (see Introduction).

The purpose of the present work was not to support any of the executive-function models in literature, but rather to explore one possible reason for the discrepancies among those models. As pointed out in the Introduction, the robustness and cogency of factor analytical approaches may suffer if there is no clear dichotomy between well-correlated and poorly correlated data sets. In absence of such a dichotomy, even small differences in the correlation pattern from different studies could substantially modify the respective best-fitting factorial models, regarding both the number and the definition of factors.

We searched for a clear dichotomy in our data but we found no distinct breakpoint between relatively low and relatively high test pair correlations. Our data therefore don’t support the view that the eight tests of the present study can be reduced to a smaller number of distinct executive functions. Instead, a more likely interpretation of our data is that executive functions form a partly overlapping structure from which our tests probed eight different regions. The overlap between the probed regions was generally low, ranging from 0.005% to 11.62% in young, and from 0.026% to 31.8% in older persons (we calculated overlap as r^2^). Of course, studies which use other tests than those in the present work will probe other regions of the postulated structure, where the overlap may be different and the resultant factorial models may therefore be different as well. This could explain why some studies found a general executive function while others did not, why some studies found a common switching-and-updating function while others did not, and why some studies found two, others three, and yet others four executive functions.

The proposed partial overlap of executive functions could also explain the wide range of model fits observed in literature. CFA yielded satisfactory loadings (>0.7) for anywhere between 17% of the administered tests [14] and 78% of those tests [13], with our own data well within that range (young: 75%; older: 50%). The use of different tests and therefore the probing of differently overlapping regions could explain this diversity of model fits. Furthermore, the proposed partial overlap is in accordance with Luria’s notion that higher-level cognition is the result of integrated activity in a distributed neural network, not of local activities in specialized modules [61]. In other words, different executive function tests possibly engaged different, slightly overlapping regions within such a network. Luria’s notion could also explain the substantial correlations observed between executive function tests on one side, and tests of fluid intelligence, memory and attention on the other side [62,63].

To our knowledge, ours is the first study to compare the structure of executive functions in young and older individuals using the same set of tests, instructions, settings and investigators. We found that correlations were significantly higher in the older group, as predicted by the concept of age-related dedifferentiation [23,22,21,25]. We further found that the pattern of relatively high and relatively low test pair correlations was not comparable in the two age groups, which is in accordance with the concept of age-related reorganization [28,27]. Thus with advancing age, larger, more overlapping, and partly different brain areas may be activated to accomplish a given task.

Several limitations of the present study should be considered. First, as in all experimental studies, our conclusions are not necessarily generalizable to persons whose education, health or activity levels differ from those in our participants (cf. Table 2), and to tests other than those administered in the present study. As a second limitation, psychomotor speed was registered by means of a stopwatch rather than electronically, and was therefore not as precise and accurate as it could have been. Third, the sample size was quite small: we analyzed data of 101 persons while others examined 100 to 486 [13,14,30,15,7]. As a fourth limitation, the number of executive function tests was small: we used two tests per putative executive function as did some earlier authors, but others used three or even four tests per putative executive function. In spite of these limitations, data from the present study are well within the range of earlier work: neither test pair correlations nor factor analytical outcomes of the present study are conspicuously different from earlier research. The unique contribution of our study therefore is not the data set collected, but rather the analytical treatment of those data.

We controlled for the effects of psychomotor speed on test outcomes [39–41] by calculating q-scores. It might be useful for future work to control for other external influences as well. For example, executive functions are probably influenced by a person’s physical, emotional and social needs [64] and controlling for those needs might therefore increase test pair correlations and accentuate age differences.

It is interesting to note that the age-related decline of executive functions was substantial in our work, as it was in earlier studies. Across all tests, q-scores were as much as 19% higher in our older compared to the young participants (cf. Fig 1). Nevertheless, all our participants lived independently in the community, arrived for testing at the agreed-upon time in the agreed-upon place and were able to follow our instructions without noticeable problems. In other words, older participants functioned well in everyday life, in spite of their deficits on standardized executive function tests. This casts doubt on the ecological validity [65,66] of such tests. Possibly, the present study and pertinent earlier work addressed phenomena which are of theoretical relevance but play only a limited role in normal life.

## Supporting information

S1 File Plos One supporting information. (XLSX)
